# Human immunodeficiency virus/acquired immunodeficiency syndrome epidemic in adolescents from a Brazilian metropolis (1978-2017)

**DOI:** 10.1590/0037-8682-0193-2019

**Published:** 2019-12-20

**Authors:** Stella Regina Taquette, Nádia Cristina Pinheiro Rodrigues

**Affiliations:** 1 Universidade do Estado do Rio de Janeiro, Departamento de Pediatria, Rio de Janeiro, RJ, Brasil.; 2 Universidade do Estado do Rio de Janeiro, Instituto de Medicina Social, Rio de Janeiro, RJ, Brasil.

**Keywords:** HIV-AIDS, Adolescence, Prevention & control, Health vulnerability, Public policies, Social control

## Abstract

**INTRODUCTION:**

Prevalence of human immunodeficiency virus among adolescents is increasing. This study aimed to analyze this current situation in Rio de Janeiro City.

**METHODS:**

This was a retrospective longitudinal study using secondary data from the National System of Notifiable Diseases database of cases in adolescents aged 13-19 years.

**RESULTS:**

There were 885 acquired immunodeficiency syndrome cases from 1978 to 2017 and 445 human immunodeficiency virus new cases from 2014 to 2017. Over time, sexually transmitted human immunodeficiency virus/acquired immunodeficiency syndrome cases increase.

**CONCLUSIONS:**

Human immunodeficiency virus/acquired immunodeficiency syndrome epidemic in adolescents requires novel prevention policies.

In Brazil, after a short period of incidence reduction, human immunodeficiency virus/acquired immunodeficiency syndrome (HIV/AIDS) is increasing, particularly in young men who have sex with men and pregnant women. According to the latest Epidemiological Bulletin of the Brazilian Ministry of Health on HIV/AIDS, referring to cases confirmed on June 2017, individuals between 15 and 19 years old continue to present increasing rates of AIDS incidence. According to distribution by sex, in the last 10 years, there is a reduced detection rate among women in all ages, except between 15 and 19 years old, where there was an increasing rate. It should be observed that prevalence ratio between sexes is lower in this age group compared to other age groups, from 17 men to 10 women, showing that the reduction in the current situation of epidemic feminization, observed in 2009, was lower in adolescents than other age groups. Among young individuals aged from 20 to 29 years, this ratio is 30 men for every 10 women^1^.

Homosexual males are predominantly exposed to HIV infection, and the number of HIV cases in homosexual males is increasing every year. A recent national HIV surveillance in a sample of 4176 men who have sex with men, performed in 12 municipalities in the five macroregions of Brazil, observed an HIV prevalence of 18.4%, significantly higher than the prevalence of 12.1% observed in 2009 in a similar study. Regarding age group, this study showed that the highest prevalence increase was observed in individuals aged between 15 and 19 years, with a triple increase in prevalence^2^.

It is worth mentioning that AIDS epidemic in adolescents is reaching the poorest population strata^3^. Regarding race/color, higher AIDS prevalence was observed in the Black population than in other racial groups. In the last 10 years, there was a reduced detection rate of AIDS in the White population (21.9%) and an increased detection rate in the Black population (35.7%), especially among women^1^.

Based on the current situation of AIDS in adolescents in Brazil, this study aimed to analyze the dynamics of the epidemic between individuals aged 13-19 years in the Rio de Janeiro City since its beginning to generate information that contributes to the creation of preventive interventions in this population.

We used data collected from HIV and AIDS cases in adolescents (aged 13-19 years) from the “Brazil’s Information System for Notifiable Diseases.” (SINAN) and population data from census and intercensus projections of the Brazilian Institute of Geography and Statistics. It is worth mentioning that HIV notification has only become mandatory in Brazil since 2014. Therefore, we only have these data from 2014. We calculated both AIDS and HIV incidence rates per 100,000 and AIDS mortality rate per 100,000 adolescents according to diagnostic year (or period of 10 years) and sex. For each individual case, we assessed sociodemographic information, type of transmission, and, in advanced cases, death.

We used chi-squared tests and Kruskal-Wallis tests to evaluate statistical difference between the number of cases by period according to sociodemographic information, type of transmission, and sexual preference.

Bayesian multilevel logistic models were used to estimate mortality AIDS odds in adolescents. Fix effects estimated in adjusted model were sex, educational level, and types of transmission (sexually transmitted, transmitted by the administration of injectable drugs, and transmitted by vertical and blood routes). In both models, we used random effects for neighborhood, diagnostic year, and Human Development Index.

We calculated generalized variance inflation factor and Hosmer and Lemeshow goodness of fit test. K-fold cross-validation method was used to evaluate model performance on different subsets of training data (k = 10).

The local ethics committee approved the project (CAAE nº 84670817.4.3001.5279).

There were 885 AIDS cases in adolescents (13-19 years old) from 1978 to 2017. The more recent the period, the higher the number of AIDS cases. We observed similar behavior of AIDS incidence rates, ranging from 0/100,000 in the first period to 5/100,000 in the last period (Table 1).

**TABLE 1:** Distribution of morbidity and mortality by human immunodeficiency virus/acquired immunodeficiency syndrome in adolescents over time in Rio de Janeiro City, Brazil.

	Total	Diagnostic moment				P-v
		1978-1987	1988-1997	1998-2007	2008-2017	
AIDS						
Morbidity						
n (%)	885 (100)	24 (3)	178 (20)	339 (38)	344 (39)	
Sex						0.0001
Female - n (%)	429 (49)	5 (1)	57 (14)	190 (44)	177 (41)	
Male - n (%)	456 (51)	19 (4)	121 (27)	149 (33)	167 (36)	
Ratio between sexes (men/10 women)	11	38	21	8	9	
Rate/100,000	3	0	3	5	5	
Sex						
Female - rate/100,000	3	0	2	6	5	
Male - rate/100,000	3	1	4	4	5	
Mortality						
n (%)	190 (22)	18 (10)	80 (42)	58 (30)	34 (18)	
Sex						0.001
Female - n (%)	66 (35)	2 (3)	23 (35)	32 (49)	9 (14)	
Male - n (%)	124 (65)	16 (13)	57 (46)	26 (21)	25 (20)	
Ratio between sexes (men/10 women)	19	80	25	8	28	
Rate/100,000	0	0	0	0	0	
Sex						
Female - rate/100,000	0	0	2	1	1	
Male - rate/100,000	1	1	1	1	0	
HIV infection		**2014**	**2015**	**2016**	**2017**	
n (%)	445 (100)	79 (18)	136 (31)	117 (26)	113 (25)	
Sex						0.05
Female	204 (46)	47 (23)	61 (30)	47 (23)	49 (24)	
Male	241 (54)	32 (13)	75 (31)	70 (29)	64 (27)	
Total - n (%)	445 (100)	79 (18)	136 (31)	117 (26)	113 (25)	
Ratio between sexes (men/10 women)	12	7	12	15	13	
Rate/100,000	16	12	20	17	16	
Sex						
Female	15	14	18	14	14	
Male	17	9	22	20	18	

**P-v**: p-value; ratio between sexes, number of cases/deaths in men for each 10 cases/deaths in women.

Deaths due to AIDS in adolescents decreased after 1988-1997. For females, this reduction only occurred in the last period, while for male, it occurred from 1998 to 2007. The death ratio between sexes decreased over time, ranging from 80 males per 10 females to 8 males per 10 females from the first period to the last two periods. The highest female mortality rate was observed in 1988-1997 (2/100,000), while for males, incidence rate remained constant from 1978-1987 to 1998-2007 (1/100,000) (Table 1).

Male incidence rate increased over the whole period, ranging from 1/100,000 in 1978-1987 to 5/100,000 in 2008-2017, while incidence rate in females showed a slight decrease in the last period. The ratio of AIDS cases between sexes decreased gradually until 1998-2007, ranging from 38 to 8-9 males per 10 females over the periods (Table 1).

Distribution of AIDS over time by sex showed that from the beginning of the epidemic, AIDS rates in adolescents increased, ranging from 0 to 8.75/100,000 in 2013 for females and from 0 to 7.03/100,000 in 2014 for males. After 2013 and 2014, rates begin to decrease for females and males, respectively.

The median age of AIDS cases was 18 years old in the most periods. Regarding ethnicity, we significantly identified non-White AIDS cases. The higher the educational level, the lower the number of AIDS cases. Regarding educational levels, AIDS cases increased in the last period. The time interval between AIDS diagnosis and its notification decreased over time, ranging from 16 to 2 months in the first period and the last period, respectively (Table 2).

Comparing transmission types, we observed that the number of AIDS cases transmitted through blood and sexual routes predominated in the first and last two periods, respectively. Over time, percentage of AIDS cases transmitted through sexual and vertical routes increases, while those transmitted through blood route decreases (Table 2).

**TABLE 2:** Distribution of characteristics of human immunodeficiency virus/acquired immunodeficiency syndrome cases in adolescents in Rio de Janeiro City by period.

	Total	Diagnostic moment				P-v
		1978-1987	1988-1997	1998-2007	2008-2017	
**AIDS**						
Median (IQR)						
Age	18 (3)	17 (3)	18 (2)	18 (3)	18 (3)	0.04
Interval - diagnosis/notification (months)	3 (32)	16 (96)	11 (49)	9 (57)	2 (7)	0.0001
n (%)						
**Ethnic**						
White	199 (37)	0 (0)	6 (3)	90 (45)	103 (52)	0.10
Non-White	335 (63)	3 (1)	11 (3)	119 (36)	202 (60)	
Pregnant	65 (15)	0 (0)	0 (0)	11 (17)	54 (83)	0.0001
**Educational level**						
< Elementary school	381 (57)	9 (2)	88 (23)	185 (49)	99 (26)	0.0001
Elementary school	204 (31)	7 (3)	40 (20)	68 (33)	89 (44)	
Middle/high school	80 (12)	0 (0)	3 (4)	18 (23)	59 (73)	
**Transmission**						
Blood	56 (2)	14 (24)	24 (43)	7 (13)	11 (20)	0.0001
**Type**						
Transfusion	3 (5)	0 (0)	1 (33)	2 (67)	0 (0)	0.38
Hemophilia	24 (43)	10 (42)	13 (54)	0 (0)	1 (4)	0.0001
Injectable drugs	29 (52)	4 (14)	10 (34)	5 (17)	10 (35)	0.001
**Sexual**	319 (36)	3 (1)	5 (1)	95 (30)	216 (68)	0.0001
**Type**						
Women						
Heterosexual	179 (95)	1 (1)	3 (2)	63 (34)	112 (63)	0.70
WSW	8 (4)	0 (0)	0 (0)	2 (25)	6 (75)	
Bisexual	2 (1)	0 (0)	0 (0)	0 (0)	2 (100)	
**Men**						
Heterosexual	30 (23)	0 (0)	2 (6)	11 (37)	17 (57)	0.01
MSM	90 (69)	1 (1)	0 (0)	19 (21)	70 (78)	
Bisexual	10 (8)	1 (10)	0 (0)	0 (0)	9 (90)	
*Vertical*	99 (11)	0 (0)	2 (2)	30 (30)	67 (68)	0.0001
**HIV infection**		**2014**	**2015**	**2016**	**2017**	
Median (IQR)						
Age	18 (2)	18 (2)	18 (2)	18 (2)	19 (2)	0.97
Interval - diagnosis/notification (days)	12 (88)	69 (266)	16 (113)	8 (82)	0 (14)	0.0001
n (%)						
**Ethnic**						
White	104 (26)	16 (15)	40 (39)	28 (27)	20 (19)	0.11
Non-White	295 (74)	58 (20)	78 (26)	80 (27)	79 (27)	
Pregnant	86 (42)	19 (22)	30 (35)	19 (22)	18 (21)	0.59
**Educational level**						
< Elementary school	148 (46)	29 (20)	39 (26)	42 (28)	38 (26)	0.38
Elementary school	124 (38)	24 (19)	39 (32)	30 (24)	31 (25)	
Middle/high school	51 (16)	6 (12)	22 (43)	14 (28)	9 (17)	
**Transmission**						
Blood	9 (2)	4 (45)	2 (22)	2 (22)	1 (11)	0.28
Type						
Transfusion	0 (0)	0 (0)	0 (0)	0 (0)	0 (0)	1.00
Hemophilia	0 (0)	0 (0)	0 (0)	0 (0)	0 (0)	1.00
Injectable drugs	9 (100)	4 (42)	2 (25)	2 (25)	1 (8)	0.28
**Sexual**	366 (82)	62 (17)	118 (32)	91 (25)	95 (26)	0.20
**Type**						
**Women**						
Heterosexual	164 (98)	36 (22)	54 (33)	35 (21)	39 (24)	0.35
WSW	1 (1)	0 (0)	1 (100)	0 (0)	0 (0)	
Bisexual	3 (2)	0 (0)	0 (0)	1 (33)	2 (67)	
**Men**						
Heterosexual	49 (25)	6 (11)	15 (31)	16 (33)	12 (25)	0.84
MSM	127 (64)	15 (12)	42 (33)	34 (27)	36 (28)	
Bisexual	22 (11)	5 (23)	6 (27)	5 (23)	6 (27)	
*Vertical*	35 (9)	4 (11)	9 (26)	15 (43)	7 (20)	0.18

**IQR:** interquartile range; **P-v**: p-value; **MSM**: men who have sex with men; **WSW**: women who have sex with women.

Regarding sex, for females, heterosexual females predominantly had AIDS in all periods; for males, heterosexual males predominantly had AIDS in the second period only. In the last two periods, homosexual males (men who have sex with men [MSM]) predominantly had AIDS. For both males and females with AIDS, percentage of all types of sexual transmission increases over time (Table 2).

There were 445 HIV cases in adolescents from 2014 to 2017. The highest number of HIV cases (n=136) and the highest HIV rate (20/100,000) were observed in 2015 (Table 1).

The number of HIV cases in female adolescents exceeded that of male cases in 2014. The significant ratio of HIV cases between sexes was observed in 2016, that is, 15 male cases per 10 female cases (Table 1).

The highest percentage of HIV cases for those who completed at least elementary school was observed in 2015. The time interval between HIV diagnosis and its notification decreased over time, ranging from 69 to 0 days from 2014 to 2017 (Table 2).

Comparing transmission types, we observed that in 2014-2017, the number of HIV cases transmitted sexually predominated. Until 2016, percentage of HIV cases transmitted through vertical route increases, while those transmitted through blood route decreased in the entire period (Table 2).

Adjusted analyses indicated that the odds of dying due to AIDS was two times higher in males than in females, 58% lower for those who completed elementary school, approximately 90% lower for those who were infected by sexual or vertical route, and three times higher for those infected by the administration of injectable drugs (Table 3).

**TABLE 3:** Simple and adjusted models to evaluate the adolescent risk of die by acquired immunodeficiency syndrome in Rio de Janeiro City.

	Simple models		Adjusted model	
	OR	95% CI	OR	95% CI
**Fixed effects**				
Male	2.05	1.32-3.22	2.12	1.36-3.32
Age	0.88	0.78-1.01		
Nonwhite race	0.65	0.39-1.10		
Year	0.86	0.84-0.88		
**Educational level**				
< Elementary school	1.00		1.00	
Elementary school	0.41	0.24-0.67	0.42	0.25-0.69
Middle/High school	0.27	0.09-0.65	0.37	0.13-0.94
**Transmission**				
**Blood vs. others**				
Transfusion	1.16	0.07-19.88		
Hemophilia	1.56	0.46-5.25		
Injectable drugs	2.90	1.04-7.83	3.15	1.29-7.58
*Sexual vs. others*	0.12	0.06-0.22	0.08	0.04-0.13
*Vertical vs. others*	0.25	0.07-0.75	0.11	0.03-0.33
				
**Random effects**			Mean	
Intercept (neighborhood)			18,629	1,266-67,205
Slope (year)			18,527	1,242-67,031
Slope (HDI)			18,630	1,268-67,203
Deviance			578	

**OR**: odds ratio; **CI**: confidence interval; **HDI**: Human Development Index; **Year**: diagnostic year.

Bayesian multilevel binomial models were used to estimate the mortality AIDS odds in adolescents. Both for simple and adjusted models, the response variable was the deaths. Fix effects estimated in the adjusted model were age, sex, educational level, and types of transmission (sexually transmitted, transmitted by the administration of injectable drugs, and transmitted by vertical and blood routes). In both models, we used random effects for neighborhood, diagnostic year, and HDI.

Data reveal an alarming situation as the more recent the period, the higher the number of AIDS cases, particularly in individuals with lower educational level, and the number of HIV cases in the last 3 years corresponds to greater than half of all AIDS cases diagnosed since the beginning of the epidemic by 2017. The increase in prevalence of HIV/AIDS signifies a poorly controlled HIV/AIDS epidemic, although it can also show an improvement and amplification of diagnosis. This situation is in contrary to the overall reduction in AIDS cases in several countries and signifies Brazil’s regression in coping with the epidemic^4^.

The increase of case prevalence from vertical transmission reveals late diagnosis of HIV, resulting in the improper administration of prophylaxis for vertical transmission in perinatal period and incorrect diagnosis of the disease during childhood. Such delay in HIV diagnosis in the studied population increases comorbidities of the disease and risk of HIV sexual transmission due to the lack of knowledge about seropositivity in high sexual experimentation^5^ ^,^ ^6^. Healthcare services have not been effective in establishing early diagnosis of HIV, and this is an essential measure to control AIDS. However, whether the establishment of late diagnosis for vertical transmission is appropriate should be properly assessed. In these cases, disease transmission may be due to unidentified sexual abuse or an error in filling out notification form.

Preventive actions for HIV/AIDS may not be accessible to youth. Today, many behavioral changes in youth brought about by digital media are observed. Conservative movements such as No-Party School^7^ implement imposition of silence regarding sexuality in the school environment, preventing dialogue on fundamental issues like gender, sexual orientation, sexual desire, and self-protection mechanisms. Social stigma, prejudice, and discrimination are human rights violations and barriers to disease control and prevention^8^ ^,^ ^9^.

The more we have a better understanding of behavioral patterns, risk practices, and social support networks, the better we can improve HIV prevention and control measures. More targeted and relevant HIV prevention programs for youth are urgently needed to prevent and control this epidemic. The increasing number of young males with HIV/AIDS is also a significant problem faced by several countries^10^. In the USA, 21% of all newly diagnosed HIV cases in 2016 were observed in young individuals aged 13 to 24 years, consisting of young gay and bisexual men (81%), especially young Black/African American and Hispanic/Latino^11^.

Hence, a novel and preventive intervention is required to prevent and control this epidemic. Adolescence is a period of sexual experimentation and identification. Adolescents receive a lot of sexual information from the Web, including pornography. Today, AIDS is considered a nonfatal disease. Hence, young individuals’ fear about AIDS is significantly different than to that of individuals in the past.

A better epidemiological situation of HIV/AIDS was observed in the past decade than in the current decade. Public policies in the past included significant participation of the society in preventive programs, and there were more campaigns and sexual education at schools in the past than in the present. There was an intense reduction of preventive measures aimed at these most affected populations, and government campaigns were curtailed by conservative politicians. Moreover, today, sexual freedom is significantly achieved. It is necessary to discuss issues related to sexuality and eroticism and to recognize legitimacy of all expressions of sexuality and gender. According to some individuals, condoms reduce pleasure in sexual intercourse. Pre-exposure prophylaxis is an alternative for those who cannot or do not want to use preservative consistently. Ways to achieve sexual pleasure and preventive methods are widely variable^12^. Nevertheless, this type of prevention strategy is not well established in many countries, including Brazil^13^.

The Global Burden of Disease Health Financing Collaborator Network^14^ in its study about spending on health and HIV/AIDS in 188 countries between 1995 and 2015 shows that the spending decreased from 2013 and the lower-middle-income countries have more cases and spend less per person. The global proportion spending on prevention was 19%, and in Brazil, it was 17.7%. The HIV seroprevalence in youth aged 15-19 years in countries that had good extensive support programs, significant participation of the society, and improved socioeconomic status significantly decreased^15^.

Reduction on time interval between diagnosis and notification and decrease in the number of deaths are positively reported. Taquette and colleagues^3^ (2011) revealed in the previous study, with similar secondary data from the SINAN in 2009, that in approximately half of the cases, notifications occurred greater than 6 months after diagnosis. Average time between diagnosis of disease and notification was 711 days. This delay compromises the coping strategy of this disease and may show a false profile of epidemic dynamic. Another point worth mentioning is reduction of deaths as a result of universal access to treatment and compulsory licensing of efavirenz in 2007 in Brazil.

This study was conducted using secondary individual notified data of HIV/AIDS. However, the SINAN does not totally show the real epidemic situation because of delay in notification and undernotification. This is one of the limitations of the study. Another limitation is the possibility of filling out notification forms incorrectly. A third limitation may be failure in identifying HIV transmission type, as pointed out in the high prevalence of vertical transmission in the studied group. Additionally, the study is restricted to Rio de Janeiro City only, which does not reflect the current HIV/AIDS situation in other regions of the country. However, we believe that it offers valuable alternative interventions to public policies to fight HIV/AIDS epidemic in adolescents and young individuals.
