# Erratum

**DOI:** 10.1590/0037-8682-0193b-2019

**Published:** 2020-01-10

**Revista da Sociedade Brasileira de Medicina Tropical/Journal of the Brazilian Society of Tropical Medicine**

**Title:** Human immunodeficiency virus/acquired immunodeficiency syndrome epidemic in adolescents from a Brazilian metropolis (1978-2017) 

Vol.:53:e20190193: 2020 - doi: 10.1590/0037-8682-0193-2019 - AUTHOR

**Nádia Cristina Pereira Rodrigues**

Should read:

**Nádia Cristina Pinheiro Rodrigues**

